# Activation of the Protein Kinase R–Like Endoplasmic Reticulum Kinase (PERK) Pathway of the Unfolded Protein Response after Experimental Traumatic Brain Injury and Treatment with a PERK Inhibitor

**DOI:** 10.1089/neur.2021.0001

**Published:** 2021-07-06

**Authors:** Rhys D. Brady, Stefanie Bird, Mujun Sun, Glenn R. Yamakawa, Brendan P. Major, Richelle Mychasiuk, Terence J. O'Brien, Stuart J. McDonald, Sandy R. Shultz

**Affiliations:** ^1^ Department of Neuroscience, Central Clinical School, Monash University, Melbourne, Victoria, Australia.; ^2^ Department of Medicine, The University of Melbourne, Parkville, Victoria, Australia.; ^3^ Department of Physiology, Anatomy, and Microbiology, La Trobe University, Bundoora, Victoria, Australia.

**Keywords:** eukaryotic translation initiation factor α, fluid percussion injury, GSK2606414, misfolded proteins, neurodegeneration, protein kinase RNA-like ER kinase

## Abstract

Neurodegeneration after traumatic brain injury (TBI) is increasingly recognized as a key factor contributing to poor chronic outcomes. Activation (i.e., phosphorylation) of the protein kinase R-like endoplasmic reticulum kinase (PERK) pathway has been implicated in neurodegenerative conditions with pathological similarities to TBI and may be a potential target to improve TBI outcomes. Here, we aimed to determine whether a moderate TBI would induce activation of the PERK pathway and whether treatment with the PERK inhibitor, GSK2606414, would improve TBI recovery. Male mice were administered a lateral fluid percussion injury (FPI) or sham injury and were euthanized at either 2 h, 24 h, or 1 week post-injury (*n* = 5 per injury group and time point) to assess changes in the PERK pathway. In the injured cortex, there was increased phosphorylated-PERK at 2 h post-FPI and increased phosphorylation of eukaryotic translation initiation factor α at 24 h post-FPI. We next examined the effect of acute treatment with GSK2606414 on pathological and behavioral outcomes at 4 weeks post-injury. Thus, there were a total of four groups: sham + VEH (*n* = 9); sham + GSK4606414 (*n* = 10); FPI + VEH (*n* = 9); and FPI + GSK2606414 (*n* = 9). GSK2606414 (50 mg/kg) or vehicle treatment was delivered by oral gavage beginning at 30 min post-injury, followed by two further treatments at 12-h increments. There were no significant effects of GSK2606414 on any of the outcomes assessed, which could be attributable to several reasons. For example, activation of PERK may not be a significant contributor to the neurological consequences 4 weeks post-FPI in mice. Further research is required to elucidate the role of the PERK pathway in TBI and whether interventions that target this pathway are beneficial.

## Introduction

Traumatic brain injury (TBI) is induced by mechanical forces applied to the brain and is a common consequence of vehicular accidents, slips/falls, assaults, as well as work-, sport-, and military-related injuries.^1^ It is a leading cause of death and disability worldwide, and there is currently no effective treatment known to mitigate the neurodegenerative aftermath that occurs in TBI survivors.^1^ Studies have identified the misfolding of several proteins in the outcomes of TBI.^2–9^ Proteins are synthesized and regulated in cells throughout the body and are essential for many cellular pathways. Consequently, dysfunctions involving important proteins may have significant repercussions, and the abnormal folding and accumulation of proteins, such as tau, transactive response DNA-binding protein 43, and amyloid-β, have all been implicated as neurotoxic factors in TBI.^2,3,5–7,10^

Unfolded protein response (UPR) is a stress response of the endoplasmic reticulum (ER) that is triggered by the abnormal folding of proteins and aims to alleviate cellular stress.^10^ The initial goal of the UPR is to restore proteostasis by reducing protein translation, degrading the misfolded proteins, and increasing the transcription of UPR-responsive genes.^10^ However, if homeostasis cannot be restored, the UPR will trigger apoptosis. To achieve proteostasis, the UPR involves three major signaling pathways (protein kinase R-like endoplasmic reticulum kinase [PERK]; inositol requiring enzyme 1; and activating transcription factor 6) that work in parallel. Activation of the PERK signaling cascade initially results in the repression of protein synthesis, but eventually triggers apoptosis if proteostasis is not achieved.^10,11^ Activation of inositol-requiring enzyme 1 and activating transcription factor 6 pathways promotes the upregulation of UPR-responsive genes.^10^ Of the three UPR pathways, there is growing evidence that PERK-UPR response is involved in a number of neurodegenerative conditions that share pathological similarities with TBI, including Alzheimer's disease, amyotrophic lateral sclerosis, and frontotemporal dementia.^11^

Considering the potential role of PERK-UPR response in these serious neurological conditions, therapeutic interventions that block PERK activation are now being developed and tested. For example, in pre-clinical models of frontotemporal dementia,^12^ subarachnoid haemorrhage,^13^ and prion disease,^14^ treatment with the potent and selective PERK inhibitor, GSK2606414,^15^ demonstrated neuroprotective effects. Of particular relevance, a recent study by Sen and colleagues found that GSK2606414 treatment prevented loss of dendritic spines and improved memory outcomes in mice after a focal brain injury.^16^

Although these findings are supportive of a neurotoxic role for PERK-UPR signaling in a number of conditions with pathological similarities to TBI, it is unknown whether the PERK-UPR pathway is affected, and whether GSK2606414 treatment is beneficial, in a clinically relevant model of mixed focal-diffuse TBI. Therefore, this study first characterized activation of the PERK-UPR pathway in a mouse model of TBI (i.e., the lateral fluid percussion injury [FPI]), which models focal/diffuse TBI pattern, at a number of time points post-injury. Next, we examined the effect of GSK2606414 treatment on pathological and behavioral outcomes 4 weeks after FPI.

## Methods

### Subjects

A total of 76, 10-week old, male C57BL/6 mice were obtained from the Australian Research Council (Perth, WA, Australia). Before injury, all mice were experimentally naïve and were group-housed under a 12-h/12-h light/dark light cycle with *ad libitum* access to food and water. After a sham or FPI procedure, mice were individually housed for the remainder of the study. All procedures were approved by The Florey Animal Ethics Committee (14-007UM) and were in compliance with the ARRIVE (Animal Research: Reporting of In Vivo Experiments) guidelines and the Australian Code of Practice for the Care and Use of Animals for Scientific Purposes by the Australian National Health and Medical Research Council.

### Experimental groups

To characterize the activation of the PERK-UPR pathway after TBI, a total of 35 mice were assigned to receive either an FPI (*n* = 20) or sham injury (*n* = 15) and either a 2-h recovery, 24-h recovery, or 1-week recovery. Five mice given an FPI died immediately after the injury and were therefore excluded from the study (25% mortality rate). Thus, there were a total of six groups: FPI +2-h recovery (*n* = 5); sham +2-h recovery (*n* = 5); FPI +24-h recovery (*n* = 5); sham +24-h recovery (*n* = 5); FPI +1-week recovery (*n* = 5); and sham +1-week recovery (*n* = 5).

To examine the effect of GSK2606414 treatment on FPI outcomes, a total of 41 mice were randomly assigned to receive either an FPI (*n* = 22) or sham injury (*n* = 19). Four mice died after FPI and were therefore excluded from the study (18% mortality). Mice were then assigned to receive treatment with either vehicle (VEH; 2% hydroxypropylmethyl cellulose +0.1% Tween-80 in H_2_O; Sigma-Aldrich, St. Louis, MO) or GSK2606414 (50 mg/kg suspended in VEH; Merck Millipore, Darmstadt, Germany). Thus, there were a total of four groups in the treatment study: sham + VEH treatment (*n* = 9); sham + GSK4606414 treatment (*n* = 10); FPI + VEH treatment (*n* = 9); and FPI + GSK2606414 treatment (*n* = 9). Treatments were delivered by oral gavage beginning at 30 min post-injury, followed by two further treatments at 12-h increments. Behavioral and pathological assessment to assess the effect of treatment on recovery was done 4 weeks later.

### Lateral fluid percussion injury

Procedures for lateral FPI and sham injury followed standard protocols as described previously.^8,17,18^ Briefly, mice were anesthetized with isoflurane and underwent a craniotomy (3 mm in diameter; centered between bregma and lambda; 2 mm right of midline). A plastic injury cap was sealed around the craniotomy using dental cement before the mouse was attached to the FPI instrument by the injury cap. At first response of hindlimb withdrawal, mice received a fluid pulse (1.5 atm). The mouse was immediately removed from the apparatus and the wound was sutured closed. Animals who received sham injury were subjected to the same procedure except for the release of the pendulum.

Apnea was measured from the time of injury to spontaneous breathing. Loss of consciousness was the time from injury to withdrawal response to a toe pinch. Self-righting reflex was the time from injury to the return of an upright position. In mice that received an FPI or sham injury procedures and were euthanized at 2 h, 24 h, or 1 week post-injury, FPI worsened apnea (*F*_2,29_ = 224.05, *p* < 0.0001), hindlimb reflex to pain (*F*_2,29_ = 370.42, *p* < 0.0001), and self-righting time (*F*_2,29_ = 372.57, *p* < 0.0001), compared to sham-injured mice (see Table 1A). For mice that were administered an FPI or sham injury and treated with either GSK2606414 or VEH, FPI increased apnea (*F*_1,36_ = 86.26, *p* < 0.0001), hindlimb reflex to pain (*F*_1,36_ = 224.59, *p* < 0.0001), and self-righting time (*F*_1,36_ = 289.14, *p* < 0.0001; see Table 1B). These acute injury severity measures are consistent with an FPI of moderate severity.

**Table 1.** Acute Injury Severity Measures

A)	Apnea	Hindlimb	Self-Righting
Sham 2 h (*n* = 5)	0 ± 0	0 ± 0	57.00 ± 5.47
Sham 24 h (*n* = 5)	0 ± 0	0 ± 0	55.00 ± 7.30
Sham 1 week (*n* = 5)	0 ± 0	0 ± 0	59.00 ± 8.60
FPI 2 h (*n* = 5)	26.60 ± 2.62^*^	202.00 ± 18.87^*^	320.40 ± 23.05^*^
FPI 24 h (*n* = 5)	29.00 ± 3.18^*^	223.40 ± 17.88^*^	337.20 ± 18.38^*^
FPI 1 week (*n* = 5)	27.60 ± 3.69^*^	206.40 ± 20.04^*^	325.20 ± 18.38^*^

B)	Apnea	Hindlimb	Self-Righting
Sham + VEH (*n* = 9)	0 ± 0	0 ± 0	51.67 ± 5.13
Sham + GSK2606414 (*n* = 9)	0 ± 0	0 ± 0	55.70 ± 3.67
FPI + VEH (*n* = 9)	27.22 ± 3.77^*^	212.89 ± 21.45^*^	347.67 ± 24.18^*^
FPI + GSK2606414 (*n* = 10)	30.44 ± 5.15^*^	219.33 ± 20.49^*^	345.56 ± 25.05^*^

A) Apnea, hindlimb, and self-righting times for the study investigating activation of the PERK pathway of the UPR at 2 h, 24 h, and 1 week post-injury. B) Apnea, hindlimb, and self-righting times for the study investigating the therapeutic potential of GSK2606414. FPI resulted in significantly longer times to recover from post-injury apnea, response to the hindlimb pain reflex test, and time to right themselves post-injury than sham injury (mean, SEM, and *N*; ^*^p < 0.001, two-way ANOVA).

FPI, fluid percussion injury; VEH, vehicle; PERK, protein kinase R-like endoplasmic reticulum kinase; UPR, unfolding protein response; SEM, standard error of the mean; ANOVA, analysis of variance.

### Behavioral testing

For the GSK2606414 treatment study, mice underwent behavioral testing to assess anxiety-like behavior, cognition, and motor function after their assigned 4-week recovery period. All tests were carried out by a researcher who was blinded to the experimental conditions over 4 consecutive days (day 1: elevated plus maze, rotarod training, Morris water maze; day 2: open field, rotarod, Morris water maze; day 3: rotarod, Morris water maze; day 4: Morris water maze), and mice were given a 2-h rest period between each task.

Anxiety-like behavior was assessed using an elevated plus maze, as previously described.^19^ The maze comprised four arms in a cross-shape, intersecting at 90-degree angles; two opposite elevated arms (height = 50 cm), and two intersecting platforms. Each arm was 55 cm long and 12 cm wide. An overhead video recorder was connected to a computer tracking software (EthoVision 3.0.15 Behavioral Monitoring/Analysis System; Noldus, Wageningen, The Netherlands) and recorded each 5-min trial. Testing took place between 8:00 am and 10:00 am, and the light level was 90 lx. The mouse was placed in the center of the maze, and the amount of time spent in the open and closed arms, as well as the number of times the animal entered the closed arm, was determined.

Locomotor and anxiety-like behavior were assessed using an open field test as previously described.^20,21^ Briefly, a circular field 90 cm in diameter was enclosed by a 40-cm-high wall. The light level was 90 lx, and testing took place from 8:00 am to 10:00 am. The animal was placed in the center of the field and was allowed to freely explore for 5 min. An overhead video recorder connected to the EthoVision tracking software (Noldus) recorded each trial, which was used to quantify the distance traveled throughout the trial.

As previously described, motor control was assessed using a rotarod (Harvard Apparatus, Holliston, MA).^22^ This apparatus comprised a motorized horizontal cylindrical rod (3 cm in diameter), suspended 30 cm above the platform, which was divided into four equal 5-cm sections by dividing walls (height = 10 cm). Trials took place over 3 consecutive days, with each day consisting of three trials. For each trial, the mouse was placed on the rod at a rotating speed of 4 rpm, which gradually accelerated to a maximum of 40 rpm. The time and rod speed at which the animal fell from the rod were recorded.

Spatial cognition was assessed using a Morris water maze test, as previously described.^23,24^ A circular pool (150 cm in diameter) was filled to a depth of ∼45 cm with tap water 21^0^C–23^0^C. Non-toxic white paint was added to the water to enable a sufficient contrast to the black mouse so that the tracking software could detect the animal. A clear Plexiglass circular platform (10 cm in diameter) was placed off-center in the pool, standing 0.5–1.0 cm below the water level, acting as an escape for the animal. Four simple abstract images were placed above the pool at North, South, East and West locations and were used as distal cues to assist the animal in finding the hidden platform. An overhead camera connected to the EthoVision software tracked each trial, and each mouse underwent four trials per day for 4 days. The time it took the animal to locate the platform as well as the total distance and average velocity of each trial were recorded.

### Western blotting

To characterize the activation of the PERK-UPR pathway after FPI or sham injury in mice, key proteins of interest, including PERK, phosphorylated-PERK (p-PERK), eukaryotic translation initiation factor-α (eIF2α), phosphorylated-eIF2α (p-eIF2α), cAMP response element-binding protein-2 (CREB-2), and growth arrest and DNA-damage-inducible 34 (GADD34), were analyzed with western blotting. PERK is a transmembrane protein located on the ER. p-PERK acts a sensor of ER stress, which leads to the phosphorylation of eIF2α to attenuate protein translation and induce translation of CREB-2.^16,25,26^ CREB-2 upregulates the expression of GADD34, which ultimately directs the dephosphorylation of p-eIF2α to restore protein synthesis.^26^

At the completion of each recovery period for study 1 (*n* = 5/group), and for mice randomly assigned to fresh tissue collection (*n* = 5/group) in study 2, mice were culled and the brains collected, as previously described.^3^ Brains were rapidly removed, the ipsilateral (containing the lesion) and contralateral cortex and hippocampus were collected and flash-frozen in liquid nitrogen, before being transferred to a −80^0^C freezer for storage. Frozen tissue was ground over dry ice and homogenized in 5% sodium dodecyl sulfate (SDS), then heated at 100^0^C for 5 min, before being centrifuged at 13,000*g* for 5 min at 24^0^C. The protein concentration of the supernatant of each sample was determined, before 30 μL of buffer (300 mM of Tris-HCl [pH 6.8], 30% 2-mercaptoethanol, 12% SDS, 0.005% bromophenol blue, and 20% glycerol) was added to each and stored at −20^0^C. Proteins in the samples were separated using SDS/polyacrylamide gel electrophoresis, and separated protein bands were then electroblotted onto polyvinyl difluoride membranes. Western blotting analysis of the proteins of the PERK pathway was conducted on the brain tissue with the following antibodies: anti-p-PERK (Thr 981; 1:50; sc-32577; Santa Cruz Biotechnology, Santa Cruz, CA); anti-PERK (C33E10; 1:1000; 3192; Cell Signaling Technology, Danvers, MA); anti-p-eIF2α (Ser51; 1:500; 9721; Cell Signaling); anti-eIf2α (L57A5; 1:1000; 2103; Cell Signaling Technology); anti-CREB-2 (C-20; 1:500; sc-200; Santa Cruz Biotechnology); anti-GADD34 (1:1000; 10449-1-AP; Proteintech, Rosemont, IL); and anti-GAPDH (glyceraldehyde 3-phosphate dehydrogenase; 1:1000; Cell Signaling Technology). Horseradish peroxidase–conjugated secondary antibodies of the respective animal were applied, and an enhanced chemiluminescent substrate kit (Amersham ECL Western-blotting detection reagents; Amersham Biosciences, Piscataway, NJ) was used to visualize protein expression on medical-grade x-ray film. ImageJ (National Institutes of Health, Bethesda, MD) was used for quantification.

### Immunohistochemistry

Mice from each treatment group (sham + VEH, *n* = 4; sham + GSK4606414, *n* = 5; FPI + VEH, *n* = 4; FPI + GSK2606414, *n* = 4) were randomly assigned to fixed tissue collection. These mice were transcardially perfused with phosphate-buffered saline (PBS; pH 7.2–7.4), followed by 4% paraformaldehyde in PBS. Brains were removed and fixed in 4% paraformaldehyde solution for 24 h at 4^0^C, followed by immersion in 70% ethanol. Ten, 12-μm-thick coronal sections evenly spaced (every 10th section) at the injury/craniotomy site were collected and mounted on slides. Four sections were used for neuronal nuclei (NeuN) staining, and six sections were used for cresyl violet staining/lesion analysis. All immunohistochemistry was conducted by a person blinded to experimental conditions.

For NeuN staining, sections were rehydrated in 0.01 M of PBS for 5 min and incubated in a blocking solution (10% normal donkey serum [NDS]; 0.1% Triton X-100 in 0.01 M of PBS) for 1 h at room temperature. Sections were then incubated in anti-NeuN antibody (1:1000; Millipore MAB377; Millipore, Burlington, MA) overnight at 4^0^C. Slides were washed in 0.01 M of PBS and were then incubated in Alexa Fluor^®^ 594 donkey antimouse immunoglobulin G (H + L) secondary antibody (A21203; 1:250 in 5% NDS +0.1% Triton X 100; Life Technologies, Carlsbad, CA) for 1 h. Slides were washed in 0.01 M of PBS for 5 min and were then incubated with 4′,6-diamidino-2-phenylindole (1:10,000 in 0.01 M of PBS) for 5 min. Slides were again washed in 0.01 M of PBS for 5 min and were mounted with Fluorescence Mounting Medium (Dako S3023; Dako A/S, Glostrup, Denmark).

Semiquantitative analysis of neuronal loss was performed by collecting images using a Brightfield Olympus 1X2-UCB microscope (Olympus Corporation, Tokyo, Japan) at an exposure time of 25 ms and a minimum of 142 and maximum of 200. Photomicrographs were captured from four coronal sections across the site of injury. A 20 × field of view of the ipsilateral cortex was sampled. ImageJ (National Institutes of Health) was used to count the number of neurons (particles >30 μm were counted), and the total number of neurons from the four sections were summed for each animal.

For cresyl violet staining, slides were warmed to room temperature over an hour, followed by heating at 37^0^C for 1 h to enhance adhesion to the slide. Sections were rehydrated in dH_2_O for 3 min and were then placed in cresyl violet for 10 min. Slides were then placed in 70%, 90%, and 100% ethanol consecutively, for 30 sec in each. Slides were then placed in xylene I for 5 min, followed by xylene II for 5 min. Slides were cover-slipped with DPX and were left to dry overnight. Six sections across the lesion site of each brain were imaged at 4 × magnification, using a Brightfield Olympus BX51. ImageJ (National Institutes of Health) was used to calculate the area of cortex contained within a box of set width in each image for each of the six sections. The box was 8.5 cm wide and centered at the mid-point between the tips of CA and dentate gyrus of the hippocampus. The combined area for the six sections was then calculated for each animal.

### Statistical analysis

All statistical analyses were performed using SPSS software (version 21.0; IBM Corp, Armonk, NY). Independent-samples *t*-tests were used to analyze protein expression for study 1. A repeated-measures analysis of variance (ANOVA) was used to analyze water-maze swim time and rotarod, whereby injury and treatment were set as between-subject factors and day (average daily trial) was set as the within-subjects factor. Two-way ANOVAs were used for all other analyses, whereby injury and treatment were set as the between-subject factors. Bonferroni *post hoc* tests were used, where appropriate. Statistical significance was set at *p* ≤ 0.05.

## Results

### Traumatic brain injury induces acute activation of the protein kinase R-like endoplasmic reticulum kinase pathway

**FIG. 1.**
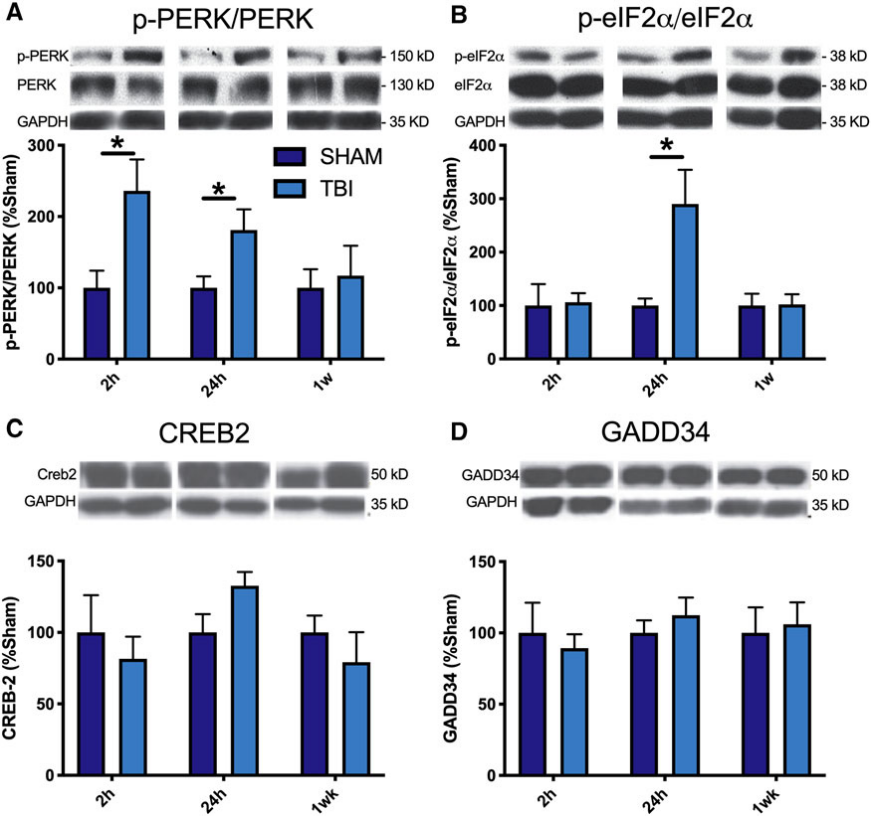
Protein expression of PERK-UPR pathway mediators in mice after TBI. Western blot analysis of the expression of (**A**) PERK-P was significantly increased at 2 and 24 h post-FPI, and (**B**) eIF2α-P was significantly decreased at 24 h post-FPI compared to sham. There were no significant differences in the expression of (**C**) CREB2 or (**D**) GADD34 between FPI and sham. Mean ± SEM. *TBI greater than sham; *p* < 0.05. CREB2, cAMP response element-binding protein-2; eIF2α, eukaryotic translation initiation factor α; FPI, fluid percussion injury; GADD34, growth arrest and DNA-damage-inducible 34; GAPDH, glyceraldehyde 3-phosphate dehydrogenase; p-eIF2α, phosphorylated eIF2α; PERK, protein kinase R-like endoplasmic reticulum kinase; p-PERK, phosphorylated PERK; SEM, standard error of the mean; TBI, traumatic brain injury; UPR, unfolded protein response.

Western blotting analysis found that FPI induced a significant increase in the ratio of p-PERK/PERK compared to sham-injured mice at 2 h (*t*_8_ = 2.84, *p* < 0.05) and 24 h (*t*_8_ = 2.38, *p* < 0.05) post-injury (see Fig. 1A). FPI mice also had increased p-eIF2α/eIF2α ratios compared to sham-injured mice at 24 h post-injury (*t*_8_ = 2.53, *p* < 0.05; see Fig. 1B). No significant differences were found between FPI- and sham-injured mice in levels of CREB-2 or GADD34 (Fig. 1C,D).

### Treatment with GSK2606414 did not reduce activation of the protein kinase R-like endoplasmic reticulum kinase pathway

**FIG. 2.**
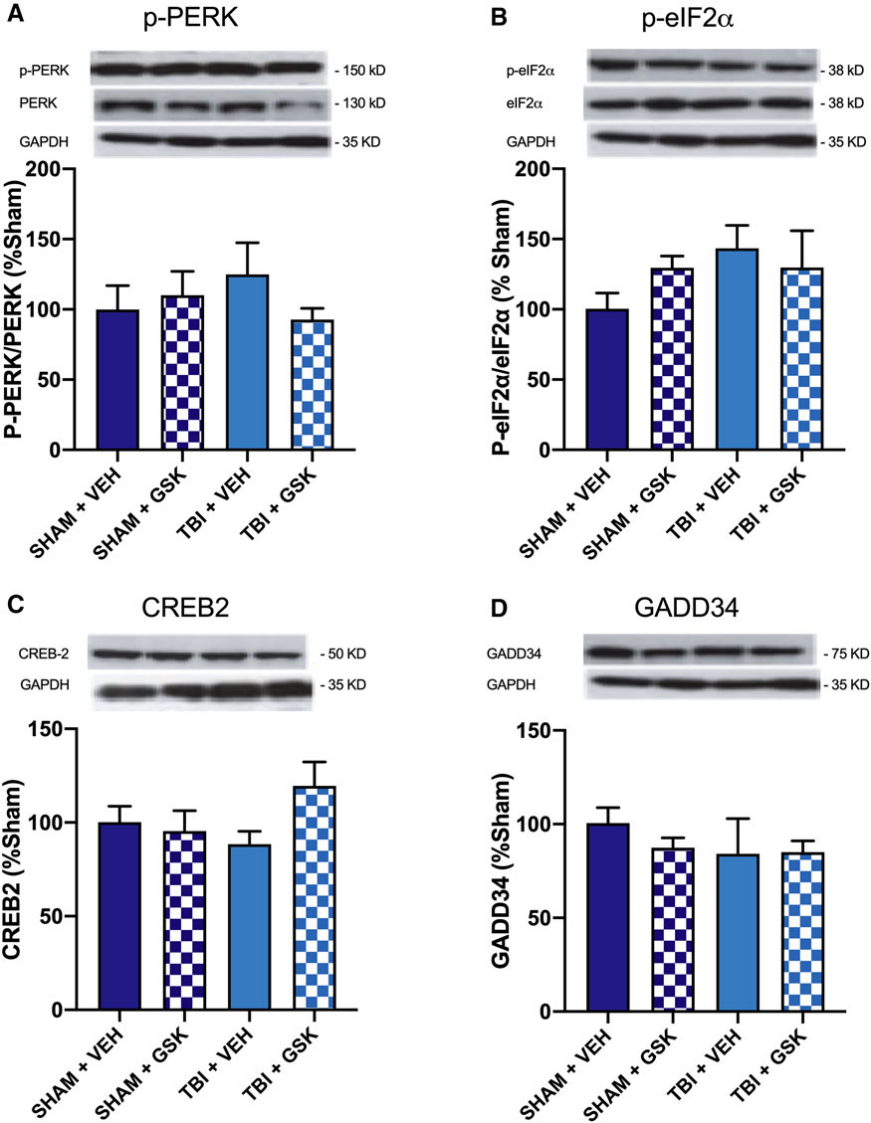
Protein expression of the mediators of the PERK-UPR pathway. Western blot analysis revealed no significant differences in the expression of (**A**) p-PERK, (**B**) p-eIF2α, (**C**) CREB2, or (**D**) GADD34 between injury or treatment groups. Mean ± SEM. CREB2, cAMP response element-binding protein-2; eIF2α, eukaryotic translation initiation factor α; GADD34, growth arrest and DNA-damage-inducible 34; GAPDH, glyceraldehyde 3-phosphate dehydrogenase; GSK, GSK2606414; p-eIF2α, phosphorylated eIF2α; PERK, protein kinase R-like endoplasmic reticulum kinase; p-PERK, phosphorylated PERK; SEM, standard error of the mean; TBI, traumatic brain injury; UPR, unfolded protein response; VEH, vehicle.

Based on the above findings, we next examined the effect of GSK2606414 treatment on long-term FPI outcomes at 4 weeks post-injury. Mice were randomly assigned to receive either an FPI or sham injury, and treatment with either VEH or GSK2606414. We found no statistically significant differences related to PERK, p-PERK, eIF2α, p-eIF2α, CREB-2, or GADD34 at 4 weeks post-injury (Fig. 2A–D).

### Treatment with GSK2606414 did not mitigate behavioral deficits after fluid percussion injury

**FIG. 3.**
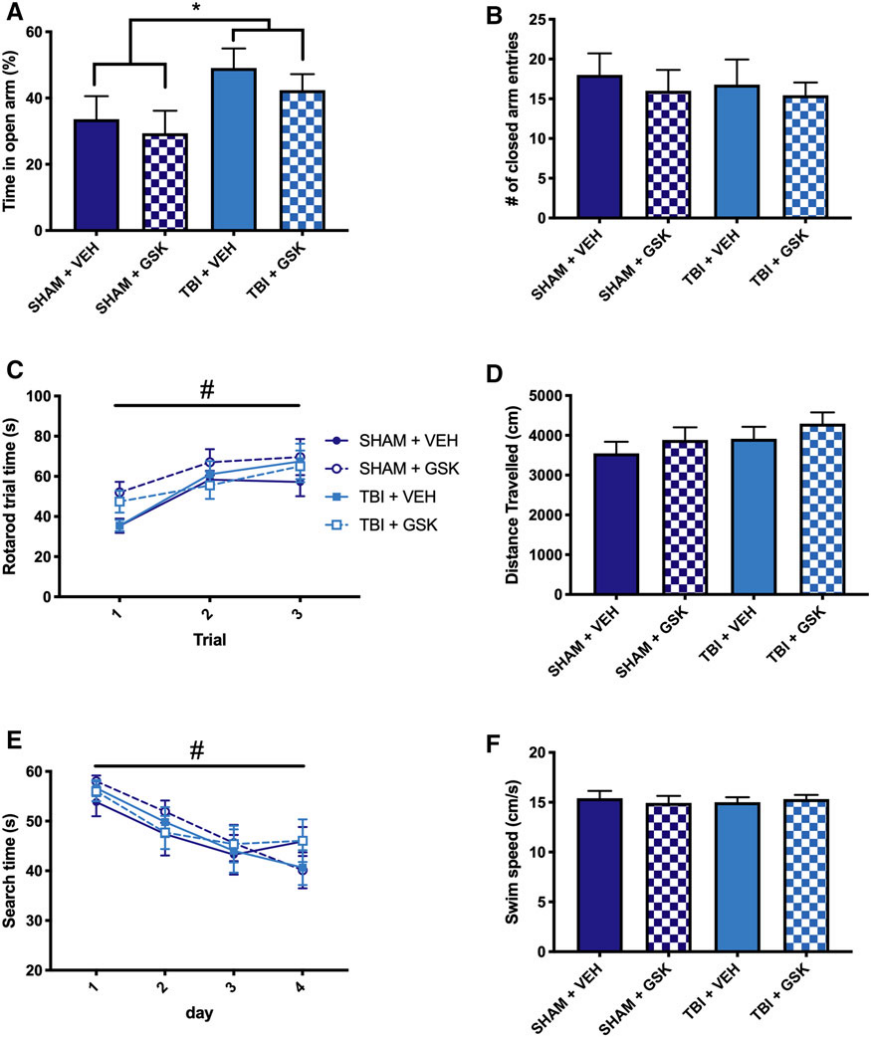
Treatment with GSK2606414 did not mitigate behavior after TBI. (**A**) FPI mice spent significantly more time in the open arms of the elevated plus maze than sham-injured mice. (**B**) There were no significant differences in the number of entries into the closed arm sections of the maze in any of the injury or treatment groups. (**C**) All mice significantly increased the amount of time spent on the rotarod before falling over the 3 days of testing. (**D**) There were no significant differences in distance traveled in the open-field in any of the injury or treatment groups. (**E**) All mice showed a significant reduction in the amount of time spent finding the hidden platform over the 4 days of water maze testing. Data are presented as an average time across the four trials for each day. (**F**) There were no differences in swim speed in any of the injury or treatment groups. Bars represent mean ± SEM. *TBI greater than sham; ^#^significant day effect; *p* < 0.05. FPI, fluid percussion injury; GSK, GSK2606414; SEM, standard error of the mean; TBI, traumatic brain injury; VEH, vehicle.

For the GSK2606414 treatment study, mice also underwent behavioral testing at 4 weeks post-injury/-treatment. Mice were tested in the elevated plus maze to assess anxiety-like behavior. A significant injury effect was found for time spent in the open arm, with FPI mice spending more time in the open arms compared to sham mice (*F*_1,33_ = 5.20, *p* < 0.05; see Fig. 3A). No significant differences were observed between treatment groups, indicating that GSK2606414 did not alter anxiety-like behavior. There were no significant group differences in the number of times mice entered the closed arms of the elevated plus maze, suggesting that the FPI-induced changes were not a result of locomotor dysfunction (see Fig. 3B).

Neuromotor function was assessed using the rotarod. Repeated-measures ANOVA revealed a significant day effect on the measure of trial time for all mice between the first day and all subsequent days (*F*_2,66_ = 22.39, *p* < 0.05; see Fig. 3C), indicating a training effect. There were no significant differences in trial time observed between any of the groups at any measured time point post-injury.

Locomotion was also assessed in the open field. There were no significant findings in distance traveled during the open field assessment (Fig. 3D), indicating no difference between the groups in locomotor ability.

Cognitive function was assessed using the water maze at 4 weeks post-injury/-treatment. There was a significant day effect on the measure of search time for all mice between the first day and all subsequent days (*F*_3,99_ = 14.35, *p* < 0.05; Fig. 3E). There were no significant group differences in search time or swim speed observed between groups (see Fig. 3E,F).

### Treatment with GSK2606414 did not alter neuronal loss or lesion volume after fluid percussion injury

**FIG. 4.**
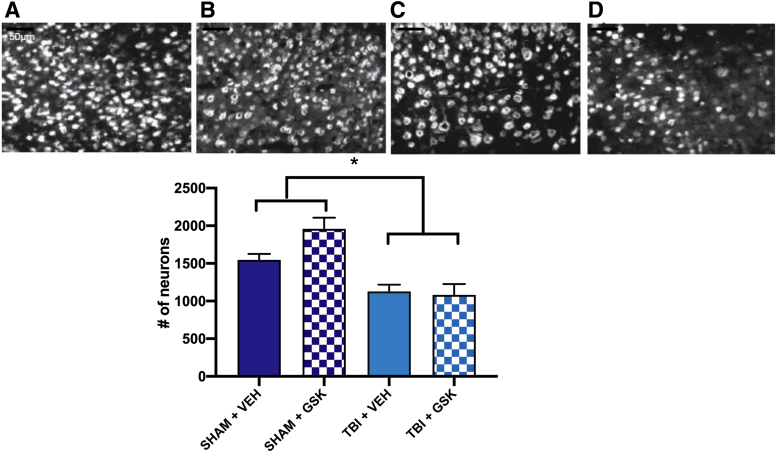
NeuN staining was used to visualize the neurons surrounding the lesion site in (**A**) Sham + VEH, (**B**) Sham + GSK2606414, (**C**) FPI + VEH, and (**D**) FPI + GSK2606414. There was a significant difference in the extent of neuronal loss observed in mice that received FPI compared to sham, but no significant differences observed between treatment groups. Bars represent mean ± SEM, *TBI less than sham; *p* < 0.05; scale bar = 50 μm. FPI, fluid percussion injury; GSK, GSK2606414; NeuN, neuronal nuclei; SEM, standard error of the mean; TBI, traumatic brain injury; VEH, vehicle.

For the GSK2606414 treatment study, the number of neurons present at the injury site was measured using a NeuN stain. Two-way ANOVA revealed a significant main effect of injury (*F*_1,13_ = 22.53, *p* < 0.05; see Fig. 4), indicating a reduction in number of neurons at the site of injury in FPI mice compared to sham mice. However, no differences in neuronal number were observed in FPI mice treated with GSK2606414 compared to FPI mice treated with vehicle.

**FIG. 5.**
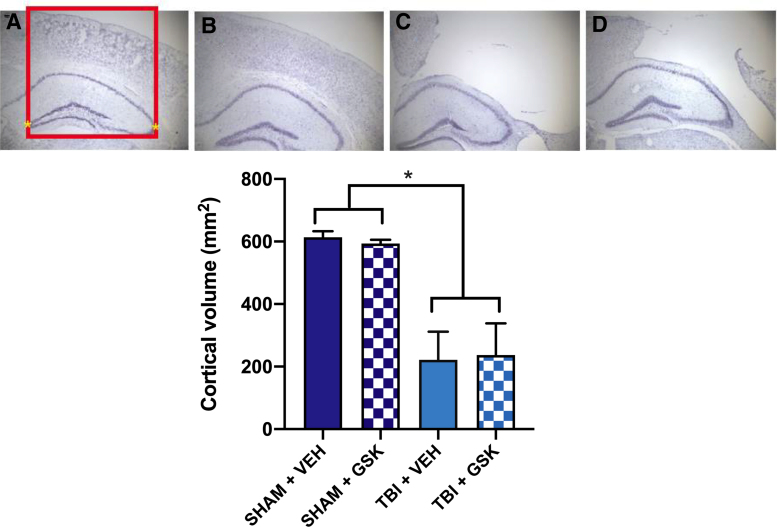
Cresyl violet staining was used to visualize the area of cortex surrounding the lesion site at in (**A**) Sham + VEH, (**B**) Sham + GSK2606414, (**C**) FPI + VEH, and (**D**) FPI + GSK2606414. Cortical area was measured within the region directly above the hippocampus (represented by the red box in A). FPI mice displayed reduced cortical volume when compared to sham mice regardless of the treatment. Bars represent mean ± SEM, *TBI less than sham; *p* < 0.05. FPI, fluid percussion injury; GSK, GSK2606414; SEM, standard error of the mean; TBI, traumatic brain injury; VEH, vehicle.

Lesion area was measured using a cresyl violet stain. Two-way ANOVA revealed a significant decrease in cortical area in FPI mice when compared to sham, regardless of which treatment was received (*F*_1,13_ = 33.77, *p* < 0.05; see Fig. 5). No difference in lesion area was observed between FPI mice treated with GSK2606414 compared to FPI mice treated with vehicle.

## Discussion

The first aim of this study was to investigate whether the PERK-UPR pathway is activated after an experimental TBI in mice. It was found that mice given an FPI had increased phosphorylation of both PERK and eIF2α acutely after injury. These findings are mostly consistent with those observed in other rodent models of brain injury, and the subtle differences regarding the temporal progression of PERK activation may be attributable to differences in severity or brain injury patterns (e.g., focal vs. diffuse).^13,16,27,28^ After FPI, which induces a mixed focal/diffuse injury pattern, we observed an acute increase in p-PERK/PERK ratio at 2 and 24 h post-TBI, which returned to baseline levels at 4 weeks post-injury. In contrast, after a controlled cortical impact (CCI), a model of focal TBI, elevated levels of p-PERK have been observed at 3 h,^27^ 12 h,^29^ 24 h,^29^ and 25 days post-injury.^16^ Activation of mediators downstream of PERK were also found to differ between injury models.^16^ Levels of p-eIF2α were increased at 25 days post-CCI,^16^ elevated from 12 to 72 h after subarachnoid haemorrhage,^13^ and raised from 4 to 48 h after intracerebral haemorrhage.^28^

In the current mouse study, we observed increased levels of p-eIF2α at 24 h post-injury and no differences at 4 weeks post-injury when compared to sham mice. In addition, chronic PERK activation results in upregulation of proapoptotic proteins, including GADD34, which has been reported 24 h after both CCI^29^ and blast TBI.^30^ However, in the current study we did not observe increased expression of GADD34 post-TBI at any time point. These findings indicate that activation of the PERK-UPR pathway may differ based on the injury pattern (focal vs. diffuse) and severity of the TBI.

The second aim of this study was to examine whether treatment with GSK2606414, a potent PERK inhibitor, would improve long-term TBI outcomes at 4 weeks post-injury. In contrast to other studies,^16^ GSK2606414 did not alter behavioral or pathological outcomes post-TBI. Post-mortem analysis revealed no effect of GSK2606414 on lesion volume or the number of neurons at the injury site. These findings may indicate that the PERK pathway does not contribute to the neurodegenerative aftermath of the mild and mixed diffuse/focal TBI used in this study to the same extent as it does to a more severe focal TBI. For example, activation of PERK appears to be extensive and prolonged after a severe focal TBI, with activation of markers increased >3.5-fold at 25 days post-CCI^16^; however, in our study, levels of PERK-UPR pathway mediators returned to baseline by 1 week post-injury. Therefore, it is likely that the beneficial effects observed by Sen and colleagues were attributable to inhibition of the chronic activation of PERK after CCI.^16,27^ These findings suggest that PERK may play a larger role in the long-term outcomes of focal brain injuries, such as penetrating ballistic injuries, when compared to milder or more diffuse injuries.

It is also possible that the incongruent findings may be attributable to methodological differences regarding the delivery of PERK inhibitors. For example, in the current study, GSK2606414 was dissolved in 2% hydroxypropylmethyl cellulose +0.1% Tween-80 in H_2_O, and the treatment was delivered by oral gavage beginning at 30 min post-injury, followed by two further treatments at 12-h increments. This regime was consistent with previous studies that demonstrated a beneficial effect of GSK2606414 treatment in pre-clinical proteopathy models.^12,14^ The 24-h treatment duration was chosen based on our initial western blotting findings, and to limit toxic effects of long-term GSK2606414 treatment that has been reported in previous studies.^12,14^ On the other hand, beneficial effects of intracerebroventricular injections of GSK2606414 dissolved in dimethyl sulfoxide were found when delivered at 1 h after intracerebral hemorrhage in rats.^28^

In addition, other studies treated with GSK2656157, a newer generation of GSK2606414 with decreased lipophilicity and improved physical properties and pharmacokinetics, and delivered the drug by an intraperitoneal injection at 30 min post-TBI followed by a daily dose for 24 days. Therefore, it is possible that a different method of administration, a different dose, timing or duration of GSK2606414 treatment, or treatment with a different PERK inhibitor may have enhanced TBI outcomes in the current study. Future studies would benefit from better understanding the pharmacological profile and functionality of GSK2606414 and establishing blood and cerebrospinal fluid levels of these PERK inhibitors. It would also be informative for future FPI studies to confirm whether the treatment protocol used in the present study influences the PERK-UPR pathway at more acute or chronic stages, given that this study only assessed the effect of GSK2606414 treatment at a single 4-week recovery time. For example, it would have been beneficial in the current study to confirm that the treatment paradigm altered the PERK-UPR pathway at 2 and 24 h post-injury (i.e., the peak of post-FPI changes).

There are other limitations that must be considered when interpreting the findings from this study. For example, the FPI model did not induce behavioral deficits on the Morris water maze or rotarod at 4 weeks post-injury, which limits the ability to detect any treatment effects on these measures. These findings are consistent with past studies,^31^ and future studies investigating whether a treatment improves long-term recovery should consider the use of alternative behavioral tests, other recovery times, or a different TBI model with more robust chronic behavioral deficits. In this study, mice were individually housed after the TBI to avoid further injury/infection to the craniotomy site attributable to interaction with other mice. This housing condition also has clinical relevance given that TBI patients can experience social isolation during recovery.^32^ However, individual housing/social isolation can influence behavior and pathological outcomes after TBI^33^ and should be considered when interpreting the current findings and designing future studies.

The order of the behavioral tasks, time of testing, and number of tasks performed are also important to consider. In the current study, the order of the tests was consistent for all mice, and more stressful tests (e.g., the water maze) were conducted last. Mice were also given a 2-h rest period between different tasks to minimize fatigue, and there were no differences found on swim speed between days of water-maze testing (i.e., swim speed did not differ if water-maze testing was or was not preceded by another test). Nonetheless, it is possible that the mice in this study grew fatigued, particularly given that their sleep cycle was disturbed by testing on repeated days (i.e., all testing was done during light hours). As such, future studies could limit behavior to a single test each day and be conducted during normal awake hours. Our study also used an abbreviated water-maze paradigm, and future studies could use more comprehensive water-maze protocols that include a visible platform trial, and additional acquisition sessions, reversal sessions, and probe sessions, to provide further insight into the cognitive deficits in mice after FPI.^34,35^ The lack of females in the present study is another limitation, particularly considering that sex differences have been reported in previous mouse FPI studies,^36^ and future studies should incorporate females.

In conclusion, the findings of this study demonstrate that although the PERK pathway is transiently activated after a focal/diffuse brain injury, oral treatment with the PERK inhibitor, GSK2606414, did not attenuate TBI-induced deficits. Taken together with other studies investigating PERK inhibition after TBI, it appears that inhibiting the PERK-UPR pathway may be more appropriate after a more severe focal TBI than a milder diffuse TBI. Our findings may also suggest that oral administration of PERK inhibitor GSK2606414 during the first 24 h post-injury may not be an optimal method for inhibiting the PERK-UPR pathway, though future studies are required to determine whether this is the case.

## Abbreviations Used

ANOVA: analysis of variance
CCI: controlled cortical impact
CREB-2: cAMP response element-binding protein-2
eIF2α: eukaryotic translation initiation factor α
ER: endoplasmic reticulum
FPI: fluid percussion injury
GADD34: growth arrest and DNA-damage-inducible 34
GAPDH: glyceraldehyde 3-phosphate dehydrogenase
NDS: normal donkey serum
NeuN: neuronal nuclei
PBS: phosphate-buffered saline
p-eIF2α: phosphorylated eIF2α
PERK: protein kinase R-like endoplasmic reticulum kinase
p-PERK: phosphorylated PERK
SDS: sodium dodecyl sulfate
TBI: traumatic brain injury
UPR: unfolded protein response
VEH: vehicle

## References

[1] Blennow, K., Hardy, J., and Zetterberg, H. (2012). The neuropathology and neurobiology of traumatic brain injury. Neuron 76, 886–899 23217738 10.1016/j.neuron.2012.11.021

[2] Kenney, K., Iacono, D., Edlow, B.L., Katz, D.I., Diaz-Arrastia, R., Dams-O'Connor, K., Daneshvar, D.H., Stevens, A., Moreau, A.L., Tirrell, L.S., Varjabedian, A., Yendiki, A., van der Kouwe, A., Mareyam, A., McNab, J.A., Gordon, W.A., Fischl, B., McKee, A.C., Perl, D.P. (2018). Dementia after moderate-severe traumatic brain injury: coexistence of multiple proteinopathies. J. Neuropathol. Exp. Neurol. 77, 50–63 29155947 10.1093/jnen/nlx101 PMC5939622

[3] Shultz, S.R., Wright, D.K., Zheng, P., Stuchbery, R., Liu, S.J., Sashindranath, M., Medcalf, R.L., Johnston, L.A., Hovens, C.M., Jones, N.C., O'Brien, T.J. (2015). Sodium selenate reduces hyperphosphorylated tau and improves outcomes after traumatic brain injury. Brain 138, 1297–1313 25771151 10.1093/brain/awv053 PMC5963409

[4] Tan, X.L., Wright, D.K., Liu, S., Hovens, C., O'Brien, T.J., and Shultz, S.R. (2016). Sodium selenate, a protein phosphatase 2A activator, mitigates hyperphosphorylated tau and improves repeated mild traumatic brain injury outcomes. Neuropharmacology 108, 382–393 27163189 10.1016/j.neuropharm.2016.05.001

[5] Johnson, V.E., Stewart, W., Trojanowski, J.Q., and Smith, D.H. (2011). Acute and chronically increased immunoreactivity to phosphorylation-independent but not pathological TDP-43 after a single traumatic brain injury in humans. Acta Neuropathol. 122, 715–726 22101322 10.1007/s00401-011-0909-9 PMC3979333

[6] Johnson, V.E., Stewart, W., and Smith, D.H. (2012). Widespread tau and amyloid-beta pathology many years after a single traumatic brain injury in humans. Brain Pathol. 22, 142–149 21714827 10.1111/j.1750-3639.2011.00513.x PMC3979351

[7] Zheng, P., Shultz, S.R., Hovens, C.M., Velakoulis, D., Jones, N.C., and O'Brien, T.J. (2014). Hyperphosphorylated tau is implicated in acquired epilepsy and neuropsychiatric comorbidities. Mol. Neurobiol. 49, 1532–1539 24323428 10.1007/s12035-013-8601-9

[8] Tan, X.L., Zheng, P., Wright, D.K., Sun, M., Brady, R.D., Liu, S., McDonald, S.J., Mychasiuk, R., Cenap, S., Jones, N.C., O'Brien, T,J., Shultz, S.R. (2020). The genetic ablation of tau improves long-term, but not short-term, functional outcomes after experimental traumatic brain injury in mice. Brain Inj. 34, 131–139 31526028 10.1080/02699052.2019.1667539

[9] Tan, X.L., Sun, M., Brady, R.D., Liu, S., Llanos, R., Cheung, S., Wright, D.K., Casillas-Espinosa, P.M., Sashindranath, M., O'Brien, T.J., McDonald, S.J., Turner, B.J., Shultz, S.R. (2019). Transactive response DNA-binding protein 43 abnormalities after traumatic brain injury. J. Neurotrauma 36, 87–99 10.1089/neu.2017.5491 29901412

[10] Scheper, W., and Hoozemans, J.J. (2015). The unfolded protein response in neurodegenerative diseases: a neuropathological perspective. Acta Neuropathol. 130, 315–331 26210990 10.1007/s00401-015-1462-8 PMC4541706

[11] Bell, M.C., Meier, S.E., Ingram, A.L., and Abisambra, J.F. (2016). PERK-opathies: an endoplasmic reticulum stress mechanism underlying neurodegeneration. Curr. Alzheimer Res. 13, 150–163 26679859 10.2174/1567205013666151218145431 PMC6542591

[12] Radford, H., Moreno, J.A., Verity, N., Halliday, M., and Mallucci, G.R. (2015). PERK inhibition prevents tau-mediated neurodegeneration in a mouse model of frontotemporal dementia. Acta Neuropathol. 130, 633–642 26450683 10.1007/s00401-015-1487-z PMC4612323

[13] Yan, F., Cao, S., Li, J., Dixon, B., Yu, X., Chen, J., Gu, C., Lin, W., Chen, G. (2017). Pharmacological inhibition of PERK attenuates early brain injury after subarachnoid hemorrhage in rats through the activation of Akt. Mol. Neurobiol. 54, 1808–1817 26887383 10.1007/s12035-016-9790-9

[14] Moreno, J.A., Halliday, M., Molloy, C., Radford, H., Verity, N., Axten, J.M., Ortori, C.A., Willis, A.E., Fischer, P.M., Barrett, D.A., Mallucci, G.R. (2013). Oral treatment targeting the unfolded protein response prevents neurodegeneration and clinical disease in prion-infected mice. Sci. Transl. Med. 5, 206ra138 10.1126/scitranslmed.3006767 24107777

[15] Axten, J.M., Medina, J.R., Feng, Y., Shu, A., Romeril, S.P., Grant, S.W., Li, W.H., Heerding, D.A., Minthorn, E., Mencken, T., Atkins, C., Liu, Q., Rabindran, S., Kumar, R., Hong, X., Goetz, A., Stanley, T., Taylor, J.D., Sigethy, S.D., Tomberlin, G.H., Hassell, A.M., Kahler, K.M., Shewchuk, L.M., Gampe, R.T. (2012). Discovery of 7-methyl-5-(1-{[3-(trifluoromethyl)phenyl]acetyl}-2,3-dihydro-1H-indol-5-yl)-7H-p yrrolo[2,3-d]pyrimidin-4-amine (GSK2606414), a potent and selective first-in-class inhibitor of protein kinase R (PKR)-like endoplasmic reticulum kinase (PERK). J. Med. Chem. 55, 7193–7207 22827572 10.1021/jm300713s

[16] Sen, T., Gupta, R., Kaiser, H., and Sen, N. (2017). Activation of PERK elicits memory impairment through inactivation of CREB and downregulation of PSD95 after traumatic brain injury. J. Neurosci. 37, 5900–5911 28522733 10.1523/JNEUROSCI.2343-16.2017 PMC5473207

[17] Shultz, S.R., Tan, X.L., Wright, D.K., Liu, S.J., Semple, B.D., Johnston, L., Jones, N.C., Cook, A.D., Hamilton, J.A., O'Brien, T.J. (2014). Granulocyte-macrophage colony-stimulating factor is neuroprotective in experimental traumatic brain injury. J. Neurotrauma 31, 976–983 24392832 10.1089/neu.2013.3106 PMC4012635

[18] Johnstone, M.R., Sun, M., Taylor, C.J., Brady, R.D., Grills, B.L., Church, J.E., Shultz, S.R., McDonald, S.J. (2018). Gambogic amide, a selective TrkA agonist, does not improve outcomes from traumatic brain injury in mice. Brain Inj. 32, 257–268 29227174 10.1080/02699052.2017.1394492

[19] Wright, D.K., Brady, R.D., Kamnaksh, A., Trezise, J., Sun, M., McDonald, S.J., Mychasiuk, R., Kolbe, S.C., Law, M., Johnston, L.A., O'Brien, T.J., Agoston, D.V., Shultz, S.R. (2019). Repeated mild traumatic brain injuries induce persistent changes in plasma protein and magnetic resonance imaging biomarkers in the rat. Sci. Rep. 9, 14626 31602002 10.1038/s41598-019-51267-w PMC6787341

[20] Brady, R.D., Grills, B.L., Romano, T., Wark, J.D., O'Brien, T.J., Shultz, S.R., McDonald, S.J. (2016). Sodium selenate treatment mitigates reduction of bone volume following traumatic brain injury in rats. J. Musculoskelet. Neuronal Interact. 16, 369–376 27973389 PMC5259578

[21] Brady, R.D., Shultz, S.R., Sun, M., Romano, T., van der Poel, C., Wright, D.K., Wark, J.D., O'Brien, T.J., Grills, B.L., McDonald, S.J. (2016). Experimental traumatic brain injury induces bone loss in rats. J. Neurotrauma 33, 2154–2160 25686841 10.1089/neu.2014.3836

[22] Sun, M., Brady, R.D., van der Poel, C., Apted, D., Semple, B.D., Church, J.E., O'Brien, T.J., McDonald, S.J., Shultz, S.R. (2018). A concomitant muscle injury does not worsen traumatic brain injury outcomes in mice. Front. Neurol. 9, 1089 30619048 10.3389/fneur.2018.01089 PMC6297867

[23] Weitzner, D.S., Engler-Chiurazzi, E.B., Kotilinek, L.A., Ashe, K.H., and Reed, M.N. (2015). Morris water maze test: optimization for mouse strain and testing environment. J. Vis. Exp. (100), 52706 10.3791/52706 PMC4545046 26132096

[24] Minter, M.R., Moore, Z., Zhang, M., Brody, K.M., Jones, N.C., Shultz, S.R., Taylor, J.M., Crack, P.J. (2016). Deletion of the type-1 interferon receptor in APP SWE/PS1 ΔE9 mice preserves cognitive function and alters glial phenotype. Acta Neuropathol. Commun. 4, 72 27400725 10.1186/s40478-016-0341-4 PMC4940712

[25] Harding, H.P., Zhang, Y., Zeng, H., Novoa, I., Lu, P.D., Calfon, M., Sadri, N., Yun, C., Popko, B., Paules, R., Stojdl, D.F., Bell, J.C., Hettmann, T., Leiden, J.M., Ron, D. (2003). An integrated stress response regulates amino acid metabolism and resistance to oxidative stress. Mol. Cell 11, 619–633 12667446 10.1016/s1097-2765(03)00105-9

[26] Brush, M.H., Weiser, D.C., and Shenolikar, S. (2003). Growth arrest and DNA damage-inducible protein GADD34 targets protein phosphatase 1α to the endoplasmic reticulum and promotes dephosphorylation of the α subunit of eukaryotic translation initiation factor 2. Mol. Cell. Biol. 23, 1292–1303 12556489 10.1128/MCB.23.4.1292-1303.2003 PMC141149

[27] Sen, T., Saha, P., Gupta, R., Foley, L.M., Jiang, T., Abakumova, O.S., Hitchens, T.K., Sen, N. (2020). Aberrant ER stress induced neuronal-IFNβ elicits white matter injury due to microglial activation and T-cell infiltration after TBI. J. Neurosci. 40, 424–446 31694961 10.1523/JNEUROSCI.0718-19.2019 PMC6948950

[28] Meng, C., Zhang, J., Dang, B., Li, H., Shen, H., Li, X., Wang, Z. (2018). PERK pathway activation promotes intracerebral hemorrhage induced secondary brain injury by inducing neuronal apoptosis both in vivo and in vitro. Front. Neurosci. 12, 111 29541018 10.3389/fnins.2018.00111 PMC5835756

[29] Farook, J.M., Shields, J., Tawfik, A., Markand, S., Sen, T., Smith, S.B., Brann, D., Dhandapani, K.M., Sen, N. (2013). GADD34 induces cell death through inactivation of Akt following traumatic brain injury. Cell Death Dis. 4, e754–e754 23907468 10.1038/cddis.2013.280 PMC3763442

[30] Logsdon, A.F., Turner, R.C., Lucke-Wold, B.P., Robson, M.J., Naser, Z.J., Smith, K.E., Matsumoto, R.R., Huber, J.D., Rosen, C.L. (2014). Altering endoplasmic reticulum stress in a model of blast-induced traumatic brain injury controls cellular fate and ameliorates neuropsychiatric symptoms. Front. Cell. Neurosci. 8, 421 25540611 10.3389/fncel.2014.00421 PMC4261829

[31] Kokiko-Cochran, O.N., Saber, M., Puntambehar, S., Bemiller, S.M., Katsumoto, A., Lee, Y.S., Bhaskar, K., Ransohoff, R.M., Lamb, B.T. (2018). Traumatic brain injury in hTau model mice: enhanced acute macrophage response and altered long-term recovery. J. Neurotrauma 35, 73–84 28859549 10.1089/neu.2017.5203 PMC5757085

[32] Salas, C.E., Casassus, M., Rowlands, L., Pimm, S., and Flanagan, D.A.J. (2018). “Relating through sameness”: a qualitative study of friendship and social isolation in chronic traumatic brain injury. Neuropsychol. Rehabil. 28, 1161–1178 27802787 10.1080/09602011.2016.1247730

[33] Khodaie, B., Lotfinia, A.A., Ahmadia, M., Lotfinia, M., Jafarian, M., Karimzadeh, F., Coulon, P., Gorji, A. (2015). Structural and functional effects of social isolation on the hippocampus of rats with traumatic brain injury. Behav. Brain Res. 278, 55–65 25264185 10.1016/j.bbr.2014.09.034

[34] Vorhees, C.V., and Williams, M.T. (2006). Morris water maze: procedures for assessing spatial and related forms of learning and memory. Nat. Protoc. 1, 848 17406317 10.1038/nprot.2006.116 PMC2895266

[35] Tucker, L.B., Fu, A.H., and McCabe, J.T. (2016). Performance of male and female C57BL/6J mice on motor and cognitive tasks commonly used in pre-clinical traumatic brain injury research. J. Neurotrauma 33, 880–894 25951234 10.1089/neu.2015.3977 PMC4860656

[36] Newell, E.A., Todd, B.P., Luo, Z., Evans, L.P., Ferguson, P.J., and Bassuk, A.G. (2020). A mouse model for juvenile, lateral fluid percussion brain injury reveals sex-dependent differences in neuroinflammation and functional recovery. J. Neurotrauma 37, 635–646 31621484 10.1089/neu.2019.6675 PMC7045348

